# Mortality and adverse events associated with statin use in primary care patients with depression: a real-world, population-based cohort study

**DOI:** 10.1136/bmjment-2024-301035

**Published:** 2024-05-20

**Authors:** Riccardo De Giorgi, Franco De Crescenzo, Edoardo Giuseppe Ostinelli, Philip J Cowen, Catherine J Harmer, Seena Fazel, Andrea Cipriani

**Affiliations:** 1 Department of Psychiatry, University of Oxford, Oxford, UK; 2 Oxford Health NHS Foundation Trust, Oxford, UK; 3 Oxford Precision Psychiatry Lab, NIHR Oxford Health Biomedical Research Centre, Oxford, UK

**Keywords:** Depression, Adult psychiatry, Depression & mood disorders

## Abstract

**Background:**

New National Institute for Health and Care Excellence (NICE) guidance endorses the prescription of statins in larger population groups for the prevention of cardiovascular and cerebrovascular morbidity and mortality, especially in people with severe mental illness. However, the evidence base for their safety and risk/benefit balance in depression is not established.

**Objectives:**

This study aims to assess the real-world mortality and adverse events of statins in depressive disorders.

**Methods:**

Population-based, nationwide (England), between-subject, cohort study. We used electronic health records (QResearch database) of people aged 18–100 years with first-episode depression, registered with English primary care practices over January 1998–August 2020 for 12(+) months, divided into statin users versus non-users.

Primary safety outcomes included all-cause mortality and any adverse event measured at 2, 6 and 12 months. Multivariable logistic regression was employed to control for several potential confounders and calculate adjusted ORs (aORs) with 99% CIs.

**Findings:**

From over 1 050 105 patients with depression (42.64% males, mean age 43.23±18.32 years), 21 384 (2.04%) died, while 707 111 (67.34%) experienced at least one adverse event during the 12-month follow-up. Statin use was associated with lower mortality over 12 months (range aOR_2–12months_ 0.66–0.67, range 99% CI 0.60 to 0.73) and with lower adverse events over 6 months (range aOR_2–6months_ 0.90–0.96, range 99% CI 0.91 to 0.99), but not at 1 year (aOR_12months_ 0.99, 99% CI 0.96 to 1.03). No association with any other individual outcome measure (ie, any other neuropsychiatric symptoms) was identified.

**Conclusions:**

We found no evidence that statin use among people with depression increases mortality or other adverse events.

**Clinical implications:**

Our findings support the safety of updated NICE guidelines for prescribing statins in people with depressive disorders.

**WHAT IS ALREADY KNOWN ON THIS TOPIC**

- Updated guidance from the National Institute for Health and Care Excellence will expand the use of statins for the primary prevention of cardiovascular and cerebrovascular disease.
- Concerns around neuropsychiatric adverse events possibly associated with statins are a barrier to their prescription in people with mental illness.
- The evidence base supporting the safety and risk/benefit of statins in people with common mental disorders such as depression is not established.

**WHAT THIS STUDY ADDS**

- Compared with non-use, statin use is associated with lower mortality and no increased adverse events, including neuropsychiatric ones, in people with first-episode depression.

**HOW THIS STUDY MIGHT AFFECT RESEARCH, PRACTICE OR POLICY**

- Patients diagnosed with depressive disorders may benefit from statin prescription, similarly to other severe mental illnesses.

## Background

Statins are a class of cholesterol-lowering drugs licensed for the prevention and treatment of cardiovascular and cerebrovascular (CVD) morbidity and mortality.^1^ They are the most prescribed medications worldwide, and more than 70 million prescriptions are dispensed in England alone, costing around £100 million to the National Health Service per year.^2^

New draft guidance from the National Institute for Health and Care Excellence (NICE) advises that a statin can be offered for the primary prevention of CVD to people above 40 years of age with a 10-year CVD event risk of <10% calculated on QRISK3,^3^ compared with prior ≥10%.^4^ This offer should follow an informed discussion between the clinician and the patient about the risk/benefit balance of statin treatment that takes into account patient preferences^3^ and comorbidities such as mental illness.^5^ For severe mental illness (SMI)—traditionally including schizophrenia, bipolar disorder and other psychoses, the importance of addressing the elevated mortality rates due to physical health conditions is established^6^ and reflected in clinical guidelines.^3 5^ This is less clear for more common mental disorders such as depression, despite evidence of robust bidirectional association with chronic ailments including CVD^7^ and ongoing high excess deaths associated with CVD, multimorbidity and health behaviours in people with depressive disorders.^8^

The overall benefit of statins has recently been challenged by studies showing small absolute risk reductions for all-cause mortality (0–0.8%) and CVD events (0.4–1.3%) adjusted for baseline risk,^9^ which would not overweigh the burden of adverse events.^10^ Recurring concerns about adverse events associated with statin use^11^ have determined a systematic underuse of these medications—a phenomenon especially seen in people with cognitive and mental disorders.^12^ However, the associations between statin use and adverse events, particularly neuropsychiatric ones, are not established.^13 14^ A long controversy on whether lowering cholesterol could result in increased depressive symptoms and suicide^15 16^ remains unsettled. Higher depression rates following statin use have been particularly observed in people with prior cerebrovascular events.^17 18^ Preclinical evidence suggests that statin administration can impair cognitive abilities while increasing anxiety levels in animals and humans, which can be explained by complex interactions between several biological and neuropsychological mechanisms.^19^

A clearer definition of the safety profile and risk/benefit balance of statins is necessary to guide a fully informed prescription of these drugs,^20^ especially in people with depression who are more at risk of premature CVD morbidity and all-cause mortality.^7 8^ Therefore, in this real-world, population-based cohort study, we investigate the mortality and adverse events associated with statin use versus non-use in a large, highly characterised sample of primary care patients with depression followed up for 12 months.

## Methods

Protocol and full methodology are shown in online supplemental file 1.

10.1136/bmjment-2024-301035.supp1 Supplementary data

### Database

QResearch is a primary care research registry (https://qresearch.org/), with anonymised electronic healthcare records of over 35 million patients registered with 1574 general practices in England, recording demographic data (eg, age), characteristics (eg, smoking status), death records, adverse events, diagnoses and prescribed medications.

### Cohort construction

The study cohort was built according to the following inclusion/exclusion criteria to include a homogeneous population with first-episode depression (ie, no treatment-resistant depression) followed up for 12 months.

*Inclusion criteria*: patients aged 18–100 years, newly diagnosed with a depressive disorder (ie, study entry date) according to validated ‘Read codes’^21 22^ (list in online supplemental file 2), registered with eligible English general practices between 1 January 1998 and 15 August 2020 for at least 12 months.

*Exclusion criteria*: any prescription of antidepressants before the study entry date; a lifetime diagnosis of schizophrenia spectrum disorder or bipolar disorder; a diagnosis of postpartum depression within ±180 from the first diagnosis of depression; any prescription of antipsychotics, mood stabilisers or more than one antidepressant at baseline.

### Exposure and comparison groups

The exposure under investigation was the concurrent use at baseline of any statin compared with non-exposure at baseline to a statin prescription: statin users versus statin non-users (drugs licensed in the UK according to the British National Formulary; list in online supplemental file 3).

### Outcomes of interest

The primary safety outcomes included:

- All-cause mortality: proportion of participants who had died during the eligible time of observation, identified using death data recorded on their general practice record.
- Any adverse event: proportion of participants with at least one adverse event (excluding all-cause mortality, list in online supplemental file 4), according to validated ‘Read codes’,^21 22^ selected among those shown to be important to patients, carers and healthcare professionals in depression.^23^

Furthermore, we examined the proportion of participants with individual neuropsychiatric adverse events, recorded via the same ‘Read codes’ that have been shown to be a potential concern associated with statin use,^13 14^ including: any psychiatric symptom, anxiety, sleep disturbance, memory impairment, self-harm, suicidality, completed suicide. We also investigated the proportion of participants who remitted from their depressive episode, defined as a Patient Health Questionnaire (PHQ)-9 score <5 during the study period.

All outcomes were measured at 2, 6 and 12 months.

### Confounders

Based on prior QResearch studies on depression and statins,^21 22^ we considered several confounding baseline variables (ie, possible risk factors associated with both the exposure and outcomes of interest). Suspected confounders included: age at study entry; sex; body mass index (BMI); year, type and severity of depression diagnosis; deprivation status (Townsend deprivation score); geographical area; smoking status; alcohol intake; ethnic group; comorbidities (eg, coronary heart disease, stroke, diabetes); antidepressant category (selective serotonin reuptake inhibitors (SSRIs), tricyclics, monoamine oxidase inhibitors, other antidepressants) and concomitant medications (eg, anticonvulsants, hypnotics/anxiolytics, antihypertensives) at baseline (list in online supplemental file 5).

### Analysis

Baseline characteristics of the study cohort were reported with descriptive statistics.

Outcomes of interest were explored with multivariable logistic regression models, clustered by general practices, to compute ORs with 99% CIs for statin users versus statin non-users (ie, between-subject design), adjusted (aOR) for the above potential confounders. Multiple imputation by chained equations was employed for missing data: for each imputation, 10 imputed datasets were generated, and coefficient estimates across these were pooled using Rubin’s rule to calculate results for the primary full-set analysis (FSA, including imputed data).

As a sensitivity analysis, we reported results for the complete-case analysis (CCA), which does not include imputed data and only accounts for cases with no missing variables. Further, FSA data for statins were compared with those of the regression analyses for aspirin to probe whether results were non-specifically associated with another medication with comparable indication (ie, prevention and treatment of CVD) in a similar population.

All statistical analyses were conducted on Stata MP V.16.0.^24^

## Findings

### Study cohort

**Figure 1:**
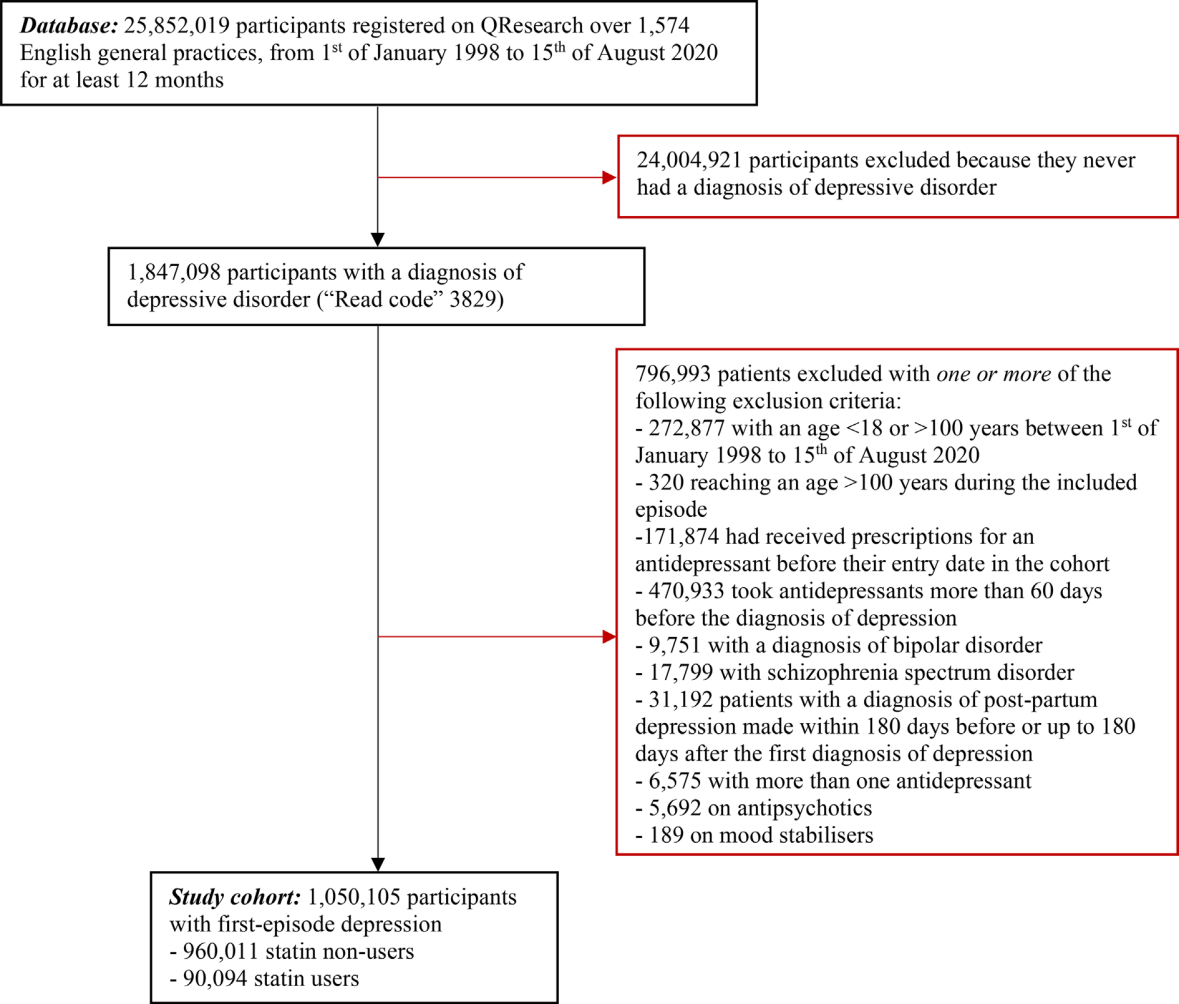
Cohort construction flow chart.

The cohort construction is in figure 1. The QResearch dataset included the electronic health records of 25 852 019 people registered with eligible general practices in England between 1 January 1998 and 15 August 2020 for at least 12 months. After the application of inclusion/exclusion criteria, the study cohort comprised 1 050 105 patients with a diagnosis of first-episode depression: 90 094 statin users and 960 011 statin non-users.

Baseline characteristics of the study cohort are in table 1. Across the whole sample, 55.06% were classified as suffering from major depression, 37.08% from minor depression and 7.86% with another ‘Read code’ for depression; the average depression severity measured with PHQ-9 at baseline was 16.15 (±5.49, moderately severe depression) and 12.94 (±6.76, moderate depression) at 12-month follow-up. The majority of the sample self-identified with white ethnicity (63.68%) and resided in London (24.13%), North West (18.52%) or South Central (11.92%) areas. The most prescribed antidepressants were SSRIs (54.94%), while antihypertensives (9.00%), hypnotics (6.68%) and contraceptives (6.00%) were the most common co-prescriptions. Compared with non-users, statin users included more males (males: 41.21% vs 57.84%), were older (mean age: 40.90 years vs 68.16 years), had more physical health comorbidities (particularly cardiovascular, cerebrovascular, metabolic and neoplastic), reported an average lower score of PHQ-9 (16.30 vs 14.33) and were taking more non-psychiatric medications (on antihypertensives: 56.52% vs 4.54%; on aspirin: 39.86% vs 2.01%; on anticoagulants: 7.71% vs 0.79%) aside from oral contraceptives (0.25% vs 6.54%), but fewer antidepressants (on antidepressants: 65.24% vs 51.99%).

**Table 1:** Baseline characteristics of the study cohort

Characteristic	Statin users N=90 094	Statin non-users N=960 011	All sample N=1 050 105
Sex			
Male	52 112 (57.84)	395 636 (41.21)	447 748 (42.64)
Mean age (SD)	68.16 (13.76)	40.90 (16.89)	43.23 (18.32)
Read codes for depression			
Major depression	34 693 (38.51)	543 520 (56.62)	578 213 (55.06)
Minor depression	34 478 (38.27)	354 900 (36.97)	389 378 (37.08)
Other	20 923 (23.22)	61 591 (6.42)	82 514 (7.86)
PHQ-9 (SD), at baseline	N=14 996	N=181 765	N=196 761
	14.33 (6.16)	16.30 (5.30)	16.15 (5.39)
At 2 months	N=6586	N=81 410	N=87 996
	11.45 (6.49)	12.43 (6.42)	12.36 (6.43)
At 6 months	N=1942	N=23 754	N=25 696
	11.13 (6.90)	12.43 (6.74)	12.34 (6.76)
At 12 months	N=1332	N=15 548	N=16 880
	11.04 (7.01)	13.10 (6.71)	12.94 (6.76)
Year of diagnosis			
1998–2005	9837 (10.92)	269 200 (28.04)	279 037 (26.57)
2006–2010	29 087 (32.29)	199 069 (20.74)	228 156 (21.73)
2011–2015	27 258 (30.26)	227 977 (23.75)	255 235 (24.31)
2016–2020	23 912 (26.54)	263 765 (27.48)	287 677 (27.40)
Body mass index (SD)	N=83 962	N=748 752	N=794 459
	28.81 (6.06)	25.90 (5.82)	26.19 (5.91)
Smoking (cigarettes/day)	N=88 601	N=886 223	N=974 824
Non-smoker	42 063 (46.69)	451 484 (47.03)	493 547 (47.00)
Ex-smoker	30 120 (33.43)	158 453 (16.51)	188 573 (17.96)
Light smoker (1–9)	15 397 (17.09)	254 227 (26.48)	269 624 (25.68)
Moderate smoker (10–19)	658 (0.73)	15 676 (1.63)	16 334 (1.56)
Heavy smoker (≥20)	363 (0.40)	6383 (0.66)	6746 (0.64)
Alcohol (daily units)	N=55 788	N=483 096	N=538 884
Non-drinker/trivial (<1)	31 348 (34.79)	268 172 (27.93)	299 520 (28.52)
Light (1–2)	16 743 (18.58)	160 061 (16.67)	176 804 (16.84)
Medium (3–6)	6229 (6.91)	41 133 (4.28)	47 362 (4.51)
Heavy (7–9)	791 (0.88)	6164 (0.64)	6955 (0.66)
Very heavy (>9)	677 (0.75)	7566 (0.79)	8243 (0.78)
Ethnic group	N=75 892	N=712 011	N=787 903
White	64 052 (71.09)	604 638 (62.98)	668 690 (63.68)
Indian	2771 (3.08)	14 280 (1.49)	17 051 (1.62)
Pakistani	1690 (1.88)	12 297 (1.28)	13 987 (1.33)
Bangladeshi	1805 (2.00)	9122 (0.95)	10 927 (1.04)
Other Asian	1452 (1.61)	11 475 (1.20)	12 927 (1.23)
Caribbean	1428 (1.59)	11 128 (1.16)	12 556 (1.20)
Black African	1033 (1.15)	14 707 (1.53)	15 740 (1.50)
Chinese	209 (0.23)	4040 (0.42)	4249 (0.40)
Other	1452 (1.61)	30 324 (3.16)	31 776 (3.03)
Townsend deprivation score in fifths	N=89 961	N=956 639	N=1 046 600
1 (least deprived)	21 498 (23.86)	189 459 (19.74)	210 957 (20.09)
2	19 795 (21.97)	195 682 (20.38)	215 477 (20.52)
3	18 073 (20.06)	196 946 (20.51)	215 019 (20.48)
4	15 992 (17.75)	188 315 (19.62)	204 307 (19.46)
5 (most deprived)	14 603 (16.21)	186 237 (19.40)	200 840 (19.13)
Region of England			
East Midlands	1896 (2.10)	35 684 (3.72)	37 580 (3.58)
East of England	2939 (3.26)	33 788 (3.52)	36 727 (3.50)
London	21 985 (24.40)	231 438 (24.11)	253 423 (24.13)
North East	3018 (3.35)	32 457 (3.38)	35 475 (3.38)
North West	19 460 (21.60)	174 967 (18.23)	194 427 (18.52)
South Central	8542 (9.48)	116 641 (12.15)	125 183 (11.92)
South East	9157 (10.16)	91 659 (9.55)	100 816 (9.60)
South West	9471 (10.51)	110 000 (11.46)	119 471 (11.38)
West Midlands	9309 (10.33)	85 637 (8.92)	94 946 (9.04)
Yorkshire & Humber	4317 (4.79)	47 740 (4.97)	52 057 (4.96)
Comorbidities at baseline			
Coronary heart disease	30 215 (33.54)	16 659 (1.74)	46 874 (4.46)
Stroke	14 949 (16.59)	14 335 (1.49)	29 284 (2.79)
Diabetes	33 644 (37.34)	28 496 (2.97)	62 140 (5.92)
Epilepsy	1906 (2.12)	13 766 (1.43)	15 672 (1.49)
Hypothyroidism	7887 (8.75)	27 221 (2.84)	35 108 (3.34)
Arthritis	22 435 (24.90)	51 393 (5.35)	73 828 (7.03)
Anxiety	8528 (9.47)	108 416 (11.29)	116 944 (11.14)
Migraine	4004 (4.44)	57 199 (5.96)	61 203 (5.83)
Cancer	17 219 (19.11)	63 390 (6.60)	80 609 (7.68)
Asthma	16 543 (18.36)	142 556 (14.85)	159 099 (15.15)
Renal failure	1820 (2.02)	2439 (0.25)	4259 (0.41)
Liver failure	2329 (2.59)	6714 (0.70)	9043 (0.86)
Osteoporosis	3739 (4.15)	9731 (1.01)	13 470 (1.28)
Suicidality	1271 (1.41)	24 497 (2.55)	25 768 (2.45)
Antidepressant category at baseline			
None	43 252 (48.01)	333 676 (34.76)	376 928 (35.89)
SSRIs	37 569 (41.70)	539 367 (56.18)	576 936 (54.94)
TCAs	3160 (3.51)	43 420 (4.52)	46 580 (4.44)
MAOIs	8 (0.01)	77 (0.01)	85 (0.01)
Other antidepressants	6105 (6.78)	43 471 (4.53)	49 576 (4.72)
Use of other drugs at baseline			
Antihypertensives	50 919 (56.52)	43 572 (4.54)	94 491 (9.00)
Aspirin	35 914 (39.86)	19 277 (2.01)	55 191 (5.26)
Anticoagulants	6945 (7.71)	7610 (0.79)	14 555 (1.39)
NSAIDs	4188 (4.65)	31 662 (3.30)	35 850 (3.41)
Anticonvulsants	3975 (4.41)	13 604 (1.42)	17 579 (1.67)
Hypnotics	6782 (7.53)	63 313 (6.60)	70 095 (6.68)
Bisphosphonates	1219 (1.35)	2407 (0.25)	3626 (0.35)
Contraceptives	224 (0.25)	62 808 (6.54)	63 032 (6.00)

Values are number of events (percentages) unless stated otherwise.

MAOIs, monoamine oxidase inhibitors; NSAIDs, non-steroidal anti-inflammatory drugs; PHQ-9, Patient Health Questionnaire-9; SSRIs, selective serotonin reuptake inhibitors; TCAs, tricyclics.

Missing data, imputed for the FSA, are in online supplemental file 6. PHQ-9 values were the least available (81.26% missing), followed by alcohol consumption (48.68% missing), ethnic group (24.97% missing) and BMI (20.70% missing).

### Study outcomes

Tables reporting the number of events for all outcomes per study group are in online supplemental file 7. A total of 21 384 (2.04%) people died, while most (67.34%, N=707 111) experienced at least one adverse event during the 12 months of study follow-up. Respectively, we observed 262 110 (24.96%) records for any psychiatric symptom, 167 274 (15.93%) for anxiety, 19 291 (1.84%) for sleep disturbance, 8524 (0.81%) for memory impairment, 6707 (0.64%) for self-harm, 16 427 (1.56%) for suicidality, 561 (0.05%) for completed suicide and 2364 (0.23%) patients remitted from the depressive episode by study endpoint.

**Figure 2:**
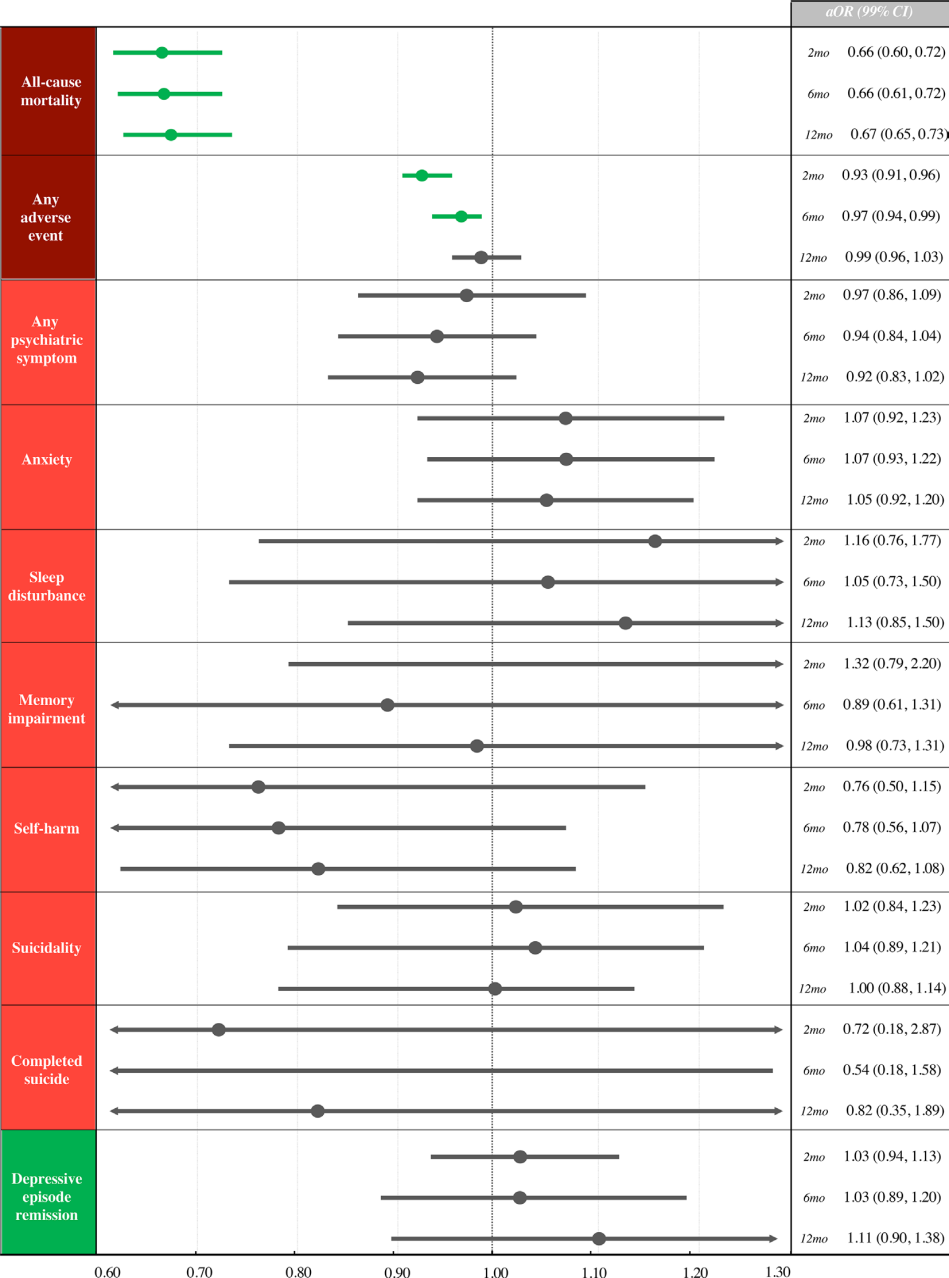
Study outcomes, full-set analyses (N=1 050 105), adjusted model. Green: beneficial association, red: harmful association, grey: no association. For depressive episode remission, an aOR >1 favours statin users, while for all outcomes, an aOR <1 favours statin users*.* 2mo, 2 months; 6mo, 6 months; 12mo, 12 months; aOR, adjusted OR.

On the FSA (N=1 050 105) adjusted model (figure 2), statin use was associated with reduced all-cause mortality at all time points (aOR_2months_ 0.66, 99% CI 0.60 to 0.72; aOR_6months_ 0.66, 99% CI 0.61 to 0.72; aOR_12months_ 0.67, 99% CI 0.65 to 0.73). A lower number of statin users experienced any adverse events at 2 and 6 months (respectively aOR_2months_ 0.93, 99% CI 0.91 to 0.96; aOR_6months_ 0.97, 99% CI 0.94 to 0.99), but not at 12 months (aOR_12months_ 0.99, 99% CI 0.96 to 1.03). No differences were identified at any time point for any psychiatric symptom (range aOR_2–12months_ 0.92–0.97), anxiety (range aOR_2–12months_ 1.05–1.07), sleep disturbance (range aOR_2–12months_ 1.05–1.16), memory impairment (range aOR_2–12months_ 0.89–1.32), self-harm (range aOR_2–12months_ 0.76–0.82), suicidality (range aOR_2–12months_ 1.00–1.02), completed suicide (range aOR_2–12months_ 0.54–0.82) or depression remission (range aOR_2–12months_ 1.03–1.11).

In statin users, results from the sensitivity analysis on the CCA (N=88 227) adjusted model also showed lower all-cause mortality (range aOR_2–12months_ 0.70–0.78) and any adverse events (range aOR_2–6months_ 0.92–0.97), but with wide CIs that crossed the line of no association. Consistently with the FSA, any psychiatric symptom (range aOR_2–12months_ 0.98–0.99), anxiety (range aOR_2–12months_ 1.02–1.04), sleep disturbance (range aOR_2–12months_ 0.95–1.02), suicidality (range aOR_2–12months_ 0.95–1.10), completed suicide (range aOR_2–12months_ 0.33–0.83) and depression remission (range aOR_2–12months_ 0.89–1.19) were not different. However, a decrease in self-harm episodes (aOR_12months_ 0.25, 99% CI 0.08 to 0.73) and higher memory impairment (aOR_12months_ 1.13, 99% CI 1.04 to 1.23) in statin users at 12 months only was observed (tables including all analyses (FSAs and CCAs, both adjusted and unadjusted) are in online supplemental file 8).

Outcomes for the sensitivity analysis on aspirin (online supplemental file 9) only partially overlapped with those of statins: aspirin use was associated with worse all-cause mortality at 12 months (aOR_12months_ 1.15, 99% CI 1.07 to 1.24) and adverse events at all time points (aOR_2months_ 1.07, 99% CI 1.07 to 1.11; aOR_6months_ 1.12, 99% CI 1.07 to 1.17; aOR_12months_ 1.18, 99% CI 1.12 to 1.24), though also fewer anxiety reports over the follow-up period (aOR_2months_ 0.90, 99% CI 0.84 to 0.96; aOR_6months_ 0.91, 99% CI 0.86 to 0.97; aOR_12months_ 0.91, 99% CI 0.85 to 0.96).

## Discussion

This study examined an array of safety outcomes associated with statins in a large cohort of 1 050 105 patients with a new diagnosis of depressive disorder followed up for 12 months in real-world conditions. The FSA-adjusted model showed that statin use, compared with non-use, was associated with lower all-cause mortality and at least comparable risk of adverse events over the follow-up period. Results differed from those of the sensitivity analysis on aspirin use, which instead displayed higher mortality rates and adverse events compared with aspirin non-use. No differences for any neuropsychiatric adverse events were observed between statin users and non-users. A sensitivity analysis of the 88 227 cases with no missing data (CCA) supported the latter lack of associations but for lower self-harm and higher memory impairment at 12 months, and was linked with lower risk of mortality and total adverse events, although not statistically significant, which was likely due to the smaller sample size.

### All-cause mortality

While the benefit of statins on all-cause mortality in the general population is being re-examined,^3 9 10^ we found a relative odds reduction between 33% and 34% across all time points in this large sample of primary care patients with depression. This finding has relevance to public health in the context of the 10–25 years of ‘mortality gap’, largely driven by physical comorbidities, in people with mental illness including depressive disorders.^7 8^ The updated tool for CVD risk scoring (QRISK3) now comprises items regarding the presence of SMI and the use of atypical antipsychotics. SMI usually involves schizophrenia, bipolar disorder and other psychoses, which tend to be the focus of CVD risk prediction studies.^25^ However, the QRISK3 development and validation cohort of people with SMI also included a high proportion of patients with moderate and severe depression (81.34%),^26^ which does not reflect the definition used in primary care electronic clinical systems.^3^ NICE concludes that QRISK3 should be used for people with ‘however defined severe mental illness’ and that clinical judgement should guide risk interpretation.^3^ Our findings that statin use is associated with reduced all-cause mortality in people with depressive disorders support the inclusion of depression within the ‘severe mental illness’ item of CVD risk prediction tools used in primary care, such as QRISK3, and call for an update of relevant UK government reports regarding the definition of physical health comorbidities and SMI,^6^ in line with other countries such as New Zealand.^25^ Specialised psychiatry services may consider implementing routine CVD risk screening for all patients diagnosed with depression within the at-risk age range of 40–74 years old,^27^ and regular cardiometabolic monitoring of individuals diagnosed with depressive disorders may be appropriate, as it is the case for other SMIs. More research is needed to understand and overcome potential barriers to statins’ uptake in people with depression.^12^

Overall, statins could helpfully treat or prevent cardiovascular and cerebrovascular conditions, thus lowering premature mortality in individuals with depression who are more at risk due to poor dietary habits and sedentary behaviour.^7 8^ In our study, this association was evident over a follow-up of 12 months, but statins are usually taken lifelong. An earlier epidemiological study of incident SSRI users (as compared with our sample of patients diagnosed with depression) with a follow-up of 642 058 person-years did not identify any association between concomitant SSRI and statin use, compared with SSRI use alone and changes in all-cause mortality (N=872 216, adjusted HR 1.04, 95% CI 0.96 to 1.12),^28^ which highlights the need for longer studies to corroborate our observations.

### Adverse events

Findings for adverse events, which were lower in statin users compared with non-users, confirm the safety of statins in the general population, while also raising the possibility of a short-term protective association in this sample of people with depression. In line with this, a meta-analysis of randomised controlled trials in depressed participants had not observed any difference between statin treatment and placebo on adverse events (N=289, relative risk 1.07, 95% CI 0.66 to 1.73).^29^

In our sample of depressed participants, we did not find evidence either confirming or refuting an association between statin use and neuropsychiatric adverse events (including anxiety, sleep disturbance, self-harm and suicidal behaviour, memory impairment) or remissions of the depressive episode. In contrast with some preclinical evidence raising concerns about possible anxiogenic properties of statins,^19^ our results for anxiety align with those of other clinical studies that had not found these deleterious effects.^30^ The commonly reported sleep abnormalities associated with statins^19^ were not seen in this subgroup of individuals diagnosed with depression, therefore more likely to experience sleep difficulties. Likewise, the lack of association between statins and self-harming or suicidal behaviour, in a population who is more susceptible to experience these, further reduces previous concerns about increased impulsivity and suicidality in statin users.^31^ In fact, the sensitivity analysis on complete cases only (CCA) suggested that self-harm at 12 months was lower in depressed subjects using statins, which is in keeping with similar results previously described in patients with schizophrenia and bipolar disorder.^32^ Similar caution should be applied to the isolated finding that memory impairment was worse for statin users on the sensitivity CCA at 12 months. While more evidence is being gathered on this matter, with not always consistent results,^14^ some concerns about the potentially harmful effects of statins on cognitive function persist.

Regarding depressive episode remission, other epidemiological studies have thus far provided conflicting results, including higher (N=7 481 000, OR 1.22, 95% CI 1.20 to 1.25)^33^ or lower (N=1 149 384, HR 0.91, 95% CI 0.88 to 0.94)^30^ depression risk in individuals taking statins. Aside from differences in their design, inconsistencies among all these findings might be explained by the selection of different study populations and endpoints as well as difficulties in controlling for possible confounders due to the lack of detailed information about the sample characteristics in the datasets. Moreover, unlike our present study, none of these other investigations had been specifically conducted on people with a primary diagnosis of depression but probed a general population of statin users.

### Limitations

Due to the observational design of this study, a lower internal validity can be anticipated as compared with an equivalent randomised controlled trial. We divided patients in an exposed and a comparison group depending on statin use at baseline; therefore, statin prescription had not been initiated at the time of entry in the study cohort (ie, when a new diagnosis of depression occurred). It could have occurred and stopped before study entry in statin non-users and may have been interrupted at any time point, as we followed an intention-to-treat principle (see protocol in online supplemental material 1)—these factors may however be responsible of selection bias. An alternative design that could be usefully employed in further studies would involve the identification of incident statin users in this population with first-episode depression and the use of a target trial emulation framework. Further, as for all analyses of electronic health records, undercoding/miscoding may occur, though the use of Read codes is established for studies on QResearch.^17^ Several baseline differences (eg, age, sex, comorbidities and concomitant medication use) could be observed between the two study cohorts, as expected for a comparison between statin users versus non-users; therefore, comparisons between these groups are challenging. However, our large and heterogeneous study population can ensure a wide overlapping across confounders, which supports the use of multiple logistic regression to control for these variables. We could not perform an additional propensity score matching analysis since this would not allow to use our imputation procedure,^34^ which may itself introduce bias. For depression severity (ie, PHQ-9 scores), alcohol use, ethnicity and BMI, a significant amount of data was missing and had to be imputed on the FSA. Specifically, PHQ-9 scores were only available for a minority of the sample (online supplemental material 6), and the large imputation procedure may have not fully captured and thus adequately adjusted for the severity of the depressive episode on the FSA. Altogether, these factors can lead to confounding by indication, which we attempted to control via multivariable logistic regressions that adjust for numerous potentially confounding variables.^21^ However, confounding variables may have differed between the physical and mental health outcomes measured. Furthermore, residual confounding remains a possibility for this study design. Because confounding cannot be completely removed, the higher short-term mortality in non-statin users versus statin users could be attributed to more deaths due to newly diagnosed depression rather than deaths due to CVDs or statins. Overall, limitations of the available database and of the study design employed advise caution in the interpretation of all our findings, and further confirmatory studies are necessary.

On the other hand, we could conduct a real-world assessment of the adverse outcomes associated with statin use in a large population of people with depression—including those with multiple comorbidities and co-occurring treatments oftentimes excluded from clinical trials, thus increasing the external validity of our results. However, statins are generally prescribed lifelong in the UK (https://www.nhs.uk/conditions/statins/); hence, it is possible that certain long-term benefits or harms would have not been observable during the 12-month follow-up. Similarly, our findings cannot be generalised beyond 12 months. We recognise that all-cause mortality measured within a 12-month time frame could be considered an uncommon occurrence; however, given the sizeable and diverse study cohort, we were still able to describe and analyse such rare event.

The primary safety outcomes (ie, all-cause mortality and proportion of participants with at least one adverse event) were assessed at three different time points (ie, at 2, 6 and 12 months) on the FSA, for a total of six main comparisons. Our assessment of the association estimates was conservative, since we used 99% CIs compared with more commonly reported 95% ones, in view of the large study sample and the several other comparisons for secondary outcomes and sensitivity analyses. Multiple comparisons might lead to an inflation of falsely positive results, but this possibility seems less relevant in the context of our findings generally showing little to no difference between the study groups on most of the additional analyses, while correcting for multiple comparisons would have spuriously lessened the chances of highlighting increases in adverse events. We did not use a time-to-event analysis, such as Cox proportional-hazard model, because the proportional-hazard assumption for the outcomes measured was not plausible when checked against available data. We used instead multiple logistic regression at prespecified time points, which does not rely on those assumptions while also allowing comparison with previous clinical trials.^29^

Our study focused on a homogeneous sample of people with a first episode of depression, often seen within primary care. It does not provide evidence, however, for those patients who have had several depressive episodes and trialled different courses of antidepressants—a condition known as ‘treatment-resistant depression’. Further research is needed to inform the prescription of statins in this population. Also, we did not assess for potentially diverse adverse event profiles associated with the use of different statins and concomitant medications. Clinical and preclinical evidence suggests that the effects of statins differ both centrally and peripherally depending on their lipophilicity^19^; therefore, our findings are not conclusive with regard to any individual statin, co-occurring drug use and their interactions.

### Clinical implications

This real-world, population-based cohort study found that statins are associated with increased adverse events or overall mortality in people with depressive disorder. Taken together, our results do not substantiate prior concerns about statin-associated adverse outcomes.^20^ Instead, they contribute to a clearer evidence base which, if supported by further randomised studies, can inform the discussion between clinicians and people with depression about the risk/benefit balance of initiating statin treatment, in agreement with updated NICE guidelines.^3^

## Data Availability

Data may be obtained from a third party and are not publicly available. To guarantee the confidentiality of personal and health information, only the authors have had access to the data during the study in accordance with the relevant licence agreements. Access to the QResearch data is according to the information on the QResearch website (https://qresearch.org/).

## References

[1] Sizar O , Khare S , Jamil RT , et al . Treasure Island (FL): StatPearls Publishing, Copyright © 2022. StatPearls Publishing LLC, 2022.

[2] O’Dowd A . NICE recommends wider use of Statins to cut cardiovascular risk. BMJ 2023;380:89. 10.1136/bmj.p89 36634930

[3] National Institute for Health and Care Excellence . Cardiovascular disease: risk assessment and reduction, including lipid modification. [NICE guideline GID-Ng10178, in development]. n.d. Available: https://www.nice.org.uk/guidance/ng238 36952507

[4] NICE guideline CG181 . National Institute for Health and Care Excellence. Cardiovascular disease: risk assessment and reduction, including lipid modification, Available: https://www.nice.org.uk/guidance/cg181 36952507

[5] National Institute for Health and Care Excellence . Multimorbidity: clinical assessment and management. [NICE guideline Ng56]. n.d. Available: https://www.nice.org.uk/guidance/ng56/

[6] National Mental Health Intelligence Network. Premature mortality in adults with severe mental illness (SMI), Available: https://www.gov.uk/government/publications/premature-mortality-in-adults-with-severe-mental-illness/premature-mortality-in-adults-with-severe-mental-illness-smi#introduction

[7] Patterson SL , Marcus M , Goetz M , et al . Depression and anxiety are associated with cardiovascular health in young adults. J Am Heart Assoc 2022;11:e027610. 10.1161/JAHA.122.027610 36533593 PMC9798786

[8] Rajan S , McKee M , Rangarajan S , et al . Association of symptoms of depression with cardiovascular disease and mortality in Low-, middle-, and high-income countries. JAMA Psychiatry 2020;77:1052–63. 10.1001/jamapsychiatry.2020.1351 32520341 PMC7287938

[9] Byrne P , Demasi M , Jones M , et al . Evaluating the association between low-density lipoprotein cholesterol reduction and relative and absolute effects of Statin treatment: A systematic review and meta-analysis. JAMA Intern Med 2022;182:474–81. 10.1001/jamainternmed.2022.0134 35285850 PMC8922205

[10] Byrne P , Cullinan J , Smith SM . Statins for primary prevention of cardiovascular disease. BMJ 2019;367:l5674. 10.1136/bmj.l5674 31619406

[11] German CA , Liao JK . Understanding the molecular mechanisms of Statin pleiotropic effects. Arch Toxicol 2023;97:1529–45. 10.1007/s00204-023-03492-6 37084080 PMC10119541

[12] Ljung R , Köster M , Björkenstam E , et al . Associations between Statin use and Suicidality, depression, anxiety, and seizures. Lancet Psychiatry 2021;8:S2215-0366(20)30512-5. 10.1016/S2215-0366(20)30512-5 33485422

[13] Giorgi R , Rizzo Pesci N , Quinton A , et al . Statins in depression: an evidence-based overview of mechanisms and clinical studies. Front Psychiatry 2021;12:702617. 34385939 10.3389/fpsyt.2021.702617 PMC8353114

[14] Samaras K , Makkar SR , Crawford JD , et al . Effects of Statins on memory, cognition, and brain volume in the elderly. J Am Coll Cardiol 2019;74:2554–68. 31753200 10.1016/j.jacc.2019.09.041

[15] Law M . Having too much evidence (depression, suicide, and low serum cholesterol). BMJ 1996;313:651–2. 10.1136/bmj.313.7058.651 8845726 PMC2351961

[16] Zureik M , Courbon D , Ducimetière P . Serum cholesterol concentration and death from suicide in men: Paris prospective study I. BMJ 1996;313:649–51. 10.1136/bmj.313.7058.649 8811757 PMC2351965

[17] Li Y , Guo Y , Zhou M , et al . Paradoxical effect of Statin medication on depressive disorder in first-ever ischemic stroke patients: possible antidepressant-like effect Prestroke and the opposite in continuous medication Poststroke. Int Clin Psychopharmacol 2021;36:147–53. 10.1097/YIC.0000000000000352 33724252

[18] Kang JH , Kao LT , Lin HC , et al . Statin use increases the risk of depressive disorder in stroke patients: a population-based study. J Neurol Sci 2015;348:89–93:S0022-510X(14)00727-8. 10.1016/j.jns.2014.11.013 25483831

[19] Giorgi R , Pesci NR , Rosso G , et al . The pharmacological bases for Repurposing Statins in depression: a review of Mechanistic studies. Translational Psychiatry 2023;13:253. 37438361 10.1038/s41398-023-02533-z PMC10338465

[20] Cai T , Abel L , Langford O , et al . Associations between Statins and adverse events in primary prevention of cardiovascular disease: systematic review with Pairwise, network, and dose-response meta-analyses. Bmj 2021;374:1537. 10.1136/bmj.n1537 PMC8279037 34261627

[21] Giorgi R , Crescenzo F , Cowen PJ , et al . Real-world outcomes of concomitant antidepressant and Statin use in primary care patients with depression: a population-based cohort study. BMC Med 2023;21:424. 37936200 10.1186/s12916-023-03138-5 PMC10631198

[22] Coupland C , Dhiman P , Morriss R , et al . Antidepressant use and risk of adverse outcomes in older people: population based cohort study. BMJ 2011;343:d4551. 10.1136/bmj.d4551 21810886 PMC3149102

[23] Chevance A , Ravaud P , Tomlinson A , et al . Identifying outcomes for depression that matter to patients, informal Caregivers, and health-care professionals: qualitative content analysis of a large International online survey. Lancet Psychiatry 2020;7:692–702:S2215-0366(20)30191-7. 10.1016/S2215-0366(20)30191-7 32711710

[24] StataCorp . Stata Statistical Software: Release 17. College Station, TX: StataCorp LLC, 2021.

[25] Cunningham R , Poppe K , Peterson D , et al . Prediction of cardiovascular disease risk among people with severe mental illness: A cohort study. PLoS One 2019;14:e0221521. 10.1371/journal.pone.0221521 31532772 PMC6750572

[26] Hippisley-Cox J , Coupland C , Brindle P . Development and validation of Qrisk3 risk prediction Algorithms to estimate future risk of cardiovascular disease: prospective cohort study. BMJ 2017;357:j2099. 10.1136/bmj.j2099 28536104 PMC5441081

[27] Public Health England . NHS health check best practice guidance: public health England. 2019 Available: https://www.healthcheck.nhs.uk/commissioners-andproviders/national-guidance/

[28] Köhler O , Gasse C , Petersen L , et al . The effect of concomitant treatment with SSRIs and Statins: A population-based study. Am J Psychiatry 2016;173:807–15. 10.1176/appi.ajp.2016.15040463 27138586

[29] Giorgi R , Waters S , Pesci NR , et al . The effects of Statin monotherapy on depressive symptoms: A systematic review and meta-analysis. J Affect Disord 2022. 10.1016/j.jad.2022.05.113 35618167

[30] Molero Y , Cipriani A , Larsson H , et al . Associations between Statin use and Suicidality, depression, anxiety, and seizures: a Swedish total-population cohort study. Lancet Psychiatry 2020;7:982–90:S2215-0366(20)30311-4. 10.1016/S2215-0366(20)30311-4 33069320 PMC7606915

[31] Kułak-Bejda A , Bejda G , Lech M , et al . Are lipids possible markers of suicide behaviors? J Clin Med 2021;10:333. 10.3390/jcm10020333 33477435 PMC7830691

[32] Hayes JF , Lundin A , Wicks S , et al . Association of Hydroxylmethyl Glutaryl coenzyme A reductase inhibitors, L-type calcium channel antagonists, and Biguanides with rates of psychiatric hospitalization and self-harm in individuals with serious mental illness. JAMA Psychiatry 2019;76:382–90. 10.1001/jamapsychiatry.2018.3907 30624557 PMC6450278

[33] Leutner M , Matzhold C , Kautzky A , et al . Major depressive disorder (MDD) and antidepressant medication are Overrepresented in high-dose Statin treatment. Front Med (Lausanne 2021;8:608083. 33644093 10.3389/fmed.2021.608083 PMC7904887

[34] Granger E , Sergeant JC , Lunt M . Avoiding pitfalls when combining multiple imputation and propensity scores. Stat Med 2019;38:5120–32. 10.1002/sim.8355 31512265 PMC6856837

