# Supplementary material for: Mortality and adverse events associated with statin use in primary care patients with depression: a real-world, population-based cohort study
Source: BMJ Ment Health. 2024 May 20;27(1):e301035. doi: 10.1136/bmjment-2024-301035 (PMC11110566; doi:10.1136/bmjment-2024-301035)
Supplement: Supplementary data [file bmjment-2024-301035supp001.pdf]

STATonly-QResearch – SUPPLEMENTARY MATERIAL

S1. Study-specific protocol

Study-specific protocol available at https://osf.io/96zcn/, STATonly-QResearch_protocol_final.pdf. Full methodology hereby reported for convenience.

METHODS

This study was independently reviewed and approved by the QResearch scientific committee (18/EM/0400), protocol in Supplementary Material, S1 (see also (1).

Database

QResearch is a primary care research registry (www.QResearch.org), with anonymised electronic healthcare records of over 35 million patients registered with 1,574 general practices in England, recording demographic data (e.g., age, gender), characteristics (e.g., height, weight, smoking status), death records, adverse events, clinical diagnoses, and prescribed medications (2).

Cohort construction

The study cohort was built according to the following inclusion/exclusion criteria to include a homogeneous population with first-episode depression (i.e., no treatment-resistant depression) followed up for 12 months. *Inclusion criteria*: patients aged 18 to 100 years, newly diagnosed with a depressive disorder (i.e., study entry date) according to validated “Read codes” (3-5) (list in Supplementary Material, S2), registered with eligible English general practices between 1st of January 1998 and 15th of August 2020 for at least 12 months. *Exclusion criteria*: any prescription of antidepressants before the study entry date; a lifetime diagnosis of schizophrenia spectrum disorder or bipolar disorder; a diagnosis of post-partum depression within 180 days before or up to 180 days after the first diagnosis of depression; any prescription of antipsychotics, mood stabilisers, or more than one antidepressant at baseline.

Exposure and comparison groups

The exposure under investigation was the concurrent use at baseline of any statin compared to non-exposure at baseline to a statin prescription: statin users *vs* statin non-users (drugs licensed in the UK according to the British National Formulary; list in Supplementary Material, S3).

Outcomes of interest

The primary safety outcomes included:
- All-cause mortality: proportion of participants who had died during the eligible time of observation, identified using death data recorded on their general practice record as per previous studies (3, 4).
- Any adverse event: proportion of participants with at least one adverse event (excluding all-cause mortality, list in Supplementary Material, S4), according to validated “Read codes” (3, 4), based on the use of preferred terms from MedDRA (https://www.meddra.org/) to categorise each adverse event into categories (1), and selected amongst those shown to be important to patients, carers, and healthcare professionals in depression (6).

Furthermore, we examined the proportion of participants with individual neuropsychiatric adverse events, recorded via the same “Read codes” (3, 4), which have been shown to be a potential concern associated to statin use (7-12), including: any psychiatric symptom, anxiety, sleep disturbance, memory impairment, self-harm, suicidality, completed suicide. We also investigated the proportion of participants who remitted from their depressive episode, defined as a Patient Health Questionnaire (PHQ)-9 score <5 (13) during the study period.

All outcomes were measured at 2 months, 6 months, and 12 months.

Confounders

Based on prior QResearch studies on depression (3, 4) and statins (5, 14), we considered several confounding baseline variables (i.e., possible risk factors associated with both the exposure and outcomes of interest). Suspected confounders included: age at study entry; sex; body mass index (BMI); year of diagnosis of depression; type of diagnosis of depression (major depressive disorders, minor depression, other); severity of index diagnosis of depression (mild, moderate, severe – based on PHQ-9 scores); deprivation status

(Townsend deprivation score) (15); geographical area of the general practice (East Midlands, East of England, London, North-East, North-West, South-Central, South-East, South-West, West Midlands, Yorkshire & Humber); smoking status (non-smoker, ex-smoker, light smoker 1-9 cigarettes/day, moderate smoker 10-19 cigarettes/day, heavy smoker ≥20 cigarettes/day, not recorded); alcohol intake (none, trivial <1 unit/day, light 1-2 unit/day, medium 3-6 unit/day, heavy 7-9 unit/day, very heavy >9 unit/day, not recorded); ethnic group (white, African-Caribbean, Asian, other); comorbidities (coronary heart disease, stroke, diabetes, hypertension, cancer, epilepsy/seizures, hypothyroidism, osteoarthritis, rheumatoid arthritis, suicidality, obesity, asthma/chronic obstructive airways disease, osteoporosis, liver disease, renal disease); antidepressant category [selective serotonin reuptake inhibitors (SSRIs), tricyclics (TCAs), monoamine oxidase inhibitors (MAOIs), other antidepressant – list in Supplementary Material, S5]; use of other drugs (anticonvulsants, hypnotics/ anxiolytics, antihypertensive drugs, aspirin, anticoagulants, non-steroidal anti-inflammatory drugs, bisphosphonates, oral contraceptives, hormone replacement therapy).

Analysis

Baseline characteristics of the study cohort were reported with descriptive statistics.

Outcomes of interest were explored with multivariable logistic regression models, clustered by general practices, to compute odds ratios (ORs) with 99% confidence intervals (99%CIs) for statin users *vs* statin non-users (i.e., between-subject design), adjusted (aOR) for the above potential confounders. Multiple imputation by chained equations was employed for missing data: for each imputation, 10 imputed datasets were generated, and coefficient estimates across these were pooled using Rubin's rule (16) to calculate results for the primary full-set analysis (FSA, including imputed data).

As a sensitivity analysis, we reported results for the complete-case analysis (CCA), which does not include imputed data and only accounts for cases with no missing variables. Further, FSA data for statins were compared with those of the regression analyses for aspirin to probe whether results were non-specifically associated with another medication with comparable indication (i.e., prevention and treatment of CVD) in a similar population.

All statistical analyses were conducted on Stata MP v16.0 (17).

Patient and public involvement and engagement

People with lived experience of unipolar depression were recruited from across the UK by the Oxford Precision Psychiatry Lab of the Oxford Health Biomedical Research Centre (https://oxfordhealthbrc.nihr.ac.uk/our-work/oxppl/patient-involvement-and-ethics/) and were involved in the design of the study (1). The drafted manuscript was critically revised by two people with lived experience and their feedback was incorporated in the final article.

CHANGES TO THE PROTOCOL

Compared to the original protocol, the current study focusses on the adverse outcomes and reports the outcome "depressive episode remission" in this context, while the other efficacy outcomes (i.e., change in depression score, response) are not described as beyond the purpose of this investigation. In any case, these two efficacy outcomes show overlapping results with the outcome "depressive episode remission" i.e., no effect.

S2. "Read codes" for depressive disorders

"Read codes" are a coded thesaurus of clinical terms used in English primary care. For depression, the relevant "Read code" is 3829.

Minor depression

Abnormal depressed feelings
Adjustment disorder with depressed mood
Adjustment reaction, predominant disturbance other emotions C/O - feeling depressed
C/O - feeling unhappy
Depressed mood
Depressive symptoms
Dysphoric mood
Emotional problem

Emotional upset
Loss of capacity for enjoyment
Loss of hope for the future
Low mood
O/E - depressed
PHQ9 score - feeling down or depressed or hopeless
Rebound mood swings
Sad mood
Stress reaction causing mixed disturbance of emotion/conduct Suspected depression
Symptoms of depression
Dysthymia

Major depression
Agitated depression
Anxiety with depression
Arteriosclerotic dementia with depression Atypical depressive disorder
Brief depressive reaction
Brief depressive reaction NOS
Chronic depression
Depressed
Depression
Depression NOS
Depression confirmed
Depressive disorder NEC
Endogenous depression
Endogenous depression - recurrent Endogenous depression first episode Masked depression
Mild depression
Moderate depression
Neurotic depression reactive type Presenile dementia with depression
Prolonged depressive reaction
Psychotic reactive depression
Reactive (neurotic) depression
Recurrent depression
Recurrent major depressive episode
Recurrent major depressive episode NOS
Recurrent major depressive episodes, unspecified
Recurrent major depressive episodes, mild
Recurrent major depressive episodes, moderate
Recurrent major depressive episodes, severe, no psychosis Recurrent major depressive episodes, severe, with psychosis Recurrent major depressive episodes, partial/unspec remission Recurrent major depressive episodes, in full remission
Senile dementia with depression
Severe depression
Single major depressive episode
Single major depressive episode NOS
Single major depressive episode, in full remission Single major depressive episode, mild
Single major depressive episode, moderate
Single major depressive episode, partial or unspec remission Single major depressive episode, severe with psychosis Single major depressive episode, severe without psychosis Single major depressive episode, unspecified
[RFC] Depression
Reactive depression NOS
Antenatal depression
Atypical depression
Depression NOS
Depressive disorder NOS
Depressive episode

Depressive episode, unspecified
Depressive neurosis
Endogenous depression with psychotic symptoms Endogenous depression without psychotic symptoms
Major depression, mild
Major depression, moderately severe
Major depression, recurrent without psychotic symptoms Major depression, severe with psychotic symptoms
Major depression, severe without psychotic symptoms
Mild anxiety depression
Mild depression
Mild depressive episode
Mixed anxiety and depressive disorder
Moderate depressive episode
Monopolar depression NOS
Neurotic depression
Other depressive episodes
Other recurrent depressive disorders
Persistant anxiety depression
Prolonged single episode of reactive depression
Recurr depress disorder cur epi severe without psyc sympt Recurr severe episodes/ major depression+psychotic symptom Recurr severe episodes/psychogenic depressive psychosis Recurrent brief depressive episodes
Recurrent depress disorder cur epi severe with psyc symp Recurrent depressive disorder
Recurrent depressive disorder, current episode mild
Recurrent depressive disorder, current episode moderate Recurrent depressive disorder, unspecified
Recurrent episodes of depressive reaction
Recurrent episodes of psychogenic depression
Recurrent episodes of reactive depression
Recurrent severe episodes of psychotic depression
Recurrent severe episodes/reactive depressive psychosis Severe depressive episode with psychotic symptoms
Severe depressive episode without psychotic symptoms
Single episode agitated depressn w'out psychotic symptoms Single episode major depression w'out psychotic symptoms Single episode of depressive reaction
Single episode of major depression and psychotic symptoms Single episode of psychogenic depression
Single episode of psychogenic depressive psychosis
Single episode of psychotic depression
Single episode of reactive depression
Single episode of reactive depressive psychosis

Other Read codes for depression
4 item geriatric depression scale
Acute posttrauma stress state
Acute stress reaction NOS
Assessment using Whooley depression screen Beck depression inventory
Beck depression inventory second edition score Depression - enhanced service completed Depression - enhanced services administration Depression annual review
Depression anxiety stress scales anxiety score Depression anxiety stress scales depression score
Depression anxiety stress scales stress score
Depression interim review
Depression management programme
Depression medication review
Depression monitoring administration
Depression monitoring first letter
Depression monitoring second letter
Depression monitoring telephone invite
Depression monitoring third letter

Depression monitoring verbal invite
Depression resolved
Depression screen
Edinburgh postnatal depression scale
Emotional and psychosocial support and advice Geriatric Depression Screen Score
Geriatric depression scale
Geriatric depression scale – 0 point
Geriatric depression scale – 1 point
Geriatric depression scale – 10 points
Geriatric depression scale – 11 points
Geriatric depression scale – 12 points
Geriatric depression scale – 13 points
Geriatric depression scale – 14 points
Geriatric depression scale – 15 points
Geriatric depression scale – 2 points
Geriatric depression scale – 3 points
Geriatric depression scale – 4 points
Geriatric depression scale – 5 points
Geriatric depression scale – 6 points
Geriatric depression scale – 7 points
Geriatric depression scale – 8 points
Geriatric depression scale – 9 points
Geriatric depression scale – <6 points
H/O: depression
HAD scale: depression score
HAMD - Hamilton rating scale for depression
HRSD - Hamilton rating scale for depression
Hospital anxiety and depression scale
MADRS - Montgomery-Asberg depression rating scale Mixed disturbance of conduct and emotion
Mood observations
On depression register
On full dose long term treatment for depression
Other acute stress reaction NOS
Other acute stress reactions
Patient given advice about management of depression Postnatal depression
Postviral depression
Referral for depression self-help video
Referral for guided self-help for depression
Removed from depression register
Stress monitoring 1st letter
[RFC] Postnatal depression
Personal history of affective disorder
Depressive personality disorder
Manic-depress psychosis,depressd, no psychotic symptoms Manic-depress psychosis,depressed type+psychotic symptoms Mood - affective disorders
Postnatal depression NOS
Postpartum depression NOS
Recurrent depressive disorder, current episode mild
Seasonal depressive disorder

S3. List of statins

Statins were included based on those currently available in the UK according to the British National Formulary (BNF) (https://bnf.nice.org.uk):
- simvastatin
- atorvastatin
- pravastatin

- fluvastatin
- rosuvastatin

S4. List of adverse events

Nausea, headache, dry mouth, insomnia, dizziness, sedation/ somnolence, diarrhoea, constipation, sexual dysfunction, fatigue, rhinitis/nasopharyngitis, hyperhidrosis, respiratory disorder (infection, cough), anxiety, decreased appetite, increased appetite, tremor, pain, vomiting, abdominal pain/discomfort, dyspepsia, agitation, visual impairment, ejaculation disorder/erectile dysfunction, weight increased, weight decreased, arrhythmia/heart rate disorder, abnormal dreams, infection, blood pressure increased, blood pressure decreased, extrapyramidal disorders, suicidal ideation, suicide behaviour or self-harm, hot flush, dysuria, skin disorder, flatulence, urinary disorders, injury, yawning, eye disorders, paraesthesia, nervous system symptoms, feeling cold, menstrual disorder, chest pain, disturbance in attention, libido increased, psychiatric symptoms, fall, confusional state, salivary hypersecretion, accidental overdose, cardiovascular symptoms (e.g., angina), sleep disturbance, oedema, aggression, completed suicide, affect lability, fever, euphoric mood, hypersomnia, memory impairment, muscular skeletal problems, serotonin syndrome, withdrawal syndrome, fractures, upper gastrointestinal bleeding, bleeding at any site, epilepsy/seizures.

S5. List of confounders and antidepressants by category

Suspected confounders included: age at study entry; sex; body mass index (BMI); year of diagnosis of depression; type of diagnosis of depression (major depressive disorders, minor depression, other); severity of index diagnosis of depression (mild, moderate, severe – based on PHQ-9 scores); deprivation status (Townsend deprivation score); geographical area of the general practice (East Midlands, East of England, London, North-East, North-West, South-Central, South-East, South-West, West Midlands, Yorkshire & Humber); smoking status (non-smoker, ex-smoker, light smoker 1-9 cigarettes/day, moderate smoker 10-19 cigarettes/day, heavy smoker ≥20 cigarettes/day, not recorded); alcohol intake (none, trivial <1 unit/day, light 1-2 unit/day, medium 3-6 unit/day, heavy 7-9 unit/day, very heavy >9 unit/day, not recorded); ethnic group (white, African-Caribbean, Asian, other); comorbidities (coronary heart disease, stroke, diabetes, hypertension, cancer, epilepsy/seizures, hypothyroidism, osteoarthritis, rheumatoid arthritis, suicidality, obesity, asthma/chronic obstructive airways disease, osteoporosis, liver disease, renal disease); antidepressant category [selective serotonin reuptake inhibitors (SSRIs), tricyclics (TCAs), monoamine oxidase inhibitors (MAOIs), other antidepressant – see below]; concomitant medications (anticonvulsants, hypnotics/ anxiolytics, antihypertensive drugs, aspirin, anticoagulants, non-steroidal anti-inflammatory drugs, bisphosphonates, oral contraceptives, hormone replacement therapy).

Antidepressants were categorised according to four classes as in the BNF:
- selective serotonin reuptake inhibitors (SSRIs): citalopram, escitalopram, fluoxetine, fluvoxamine, paroxetine, sertraline
- tricyclics (TCAs): amitriptyline, amoxapine, butriptyline, clomipramine, desipramine, dosulepin, doxepin, imipramine, iprindole, lofepramine, maprotiline, mianserin, nortriptyline, protriptyline, trimipramine, viloxazine
- monoamine oxidase inhibitors (MAOIs): isocarboaxazid, moclobemide, phenelzine, tranylcypromine
- other antidepressants: agomelatine, duloxetine, mirtazapine, nefazodone, reboxetine, tryptophan, trazodone, venlafaxine, vortioxetine

S6. Missing data

Table S5. *Missing data (percentage) imputed for the full set analysis*

Characteristic	Missing (%)	Complete
PHQ-9		

at baseline	853,344 (81.26)	1,050,105
at 2 months	962,109 (91.62)	1,050,105
at 6 months	1,024,410 (97.55)	1,050,105
at 12 months	1,033,225 (98.39)	1,050,105
Body Mass Index	217,391 (20.70)	1,050,105
Smoking	75,281 (7.17)	1,050,105
Alcohol	511,221 (48.68)	1,050,105
Ethnic group	262,202 (24.97)	1,050,105
Townsend deprivation score	3,505 (0.33)	1,050,105

S7. Number of events for all outcomes

Table S7. *Number of events (percentage) for all outcomes at 2 months, 6 months, and 12 months*

Outcome	**Statin users**			**Statin non-users**		
	Nº events	*%*	*Nº patients*	*Nº events*	*%*	*Nº patients*
**All-cause mortality**						
2mo	1,354	(1.50)	90,094	4,811	(0.50)	960,011
6mo	2,031	(2.25)	90,094	7,216	(0.75)	960,011
12mo	5,473	(6.07)	90,094	15,911	(1.66)	960,011
**Any adverse event**						
2mo	36,870	(40.92)	90,094	368,157	(38.35)	960,011
6mo	55,139	(61.20)	90,094	526,875	(54.88)	960,011
12mo	68,051	(75.53)	90,094	639,060	(66.57)	960,011
**Any psychiatric symptom**						
2mo	12,786	(14.19)	90,094	158,410	(16.50)	960,011
6mo	16,774	(18.62)	90,094	203,877	(21.24)	960,011
12mo	20,534	(22.79)	90,094	241,576	(25.16)	960,011
**Anxiety**						
2mo	6,264	(6.95)	90,094	111,440	(11.61)	960,011
6mo	7,657	(8.50)	90,094	138,129	(14.49)	960,011
12mo	8,730	(9.69)	90,094	158,544	(16.51)	960,011
**Sleep disturbance**						
2mo	619	(0.69)	90,094	5,906	(0.62)	960,011
6mo	1,250	(1.39)	90,094	11,289	(1.18)	960,011
12mo	2,043	(2.27)	90,094	17,248	(1.80)	960,011
**Memory impairment**						
2mo	771	(0.86)	90,094	1,738	(0.18)	960,011
6mo	1,537	(1.71)	90,094	3,673	(0.38)	960,011
12mo	2,543	(2.82)	90,094	5,981	(0.62)	960,011
**Self-harm**						
2mo	73	(0.08)	90,094	2,636	(0.27)	960,011
6mo	123	(0.14)	90,094	4,758	(0.50)	960,011
12mo	169	(0.19)	90,094	6,538	(0.68)	960,011

**Suicidality**						
2mo	448	(0.50)	90,094	7,453	(0.78)	960,011
6mo	702	(0.78)	90,094	11,814	(1.23)	960,011
12mo	930	(1.03)	90,094	15,497	(1.61)	960,011
**Completed suicide**						
2mo	7	(0.01)	90,094	230	(0.02)	960,011
6mo	10	(0.01)	90,094	389	(0.04)	960,011
12mo	17	(0.02)	90,094	544	(0.06)	960,011
**Depressive episode remission**						
2mo	1,144	(17.37)	6,586	10,693	(13.13)	81,410
6mo	450	(23.17)	1,942	3,708	(15.61)	23,753
12mo	314	(23.57)	1,332	2,050	(13.18)	15,548

S8. Full set analyses (FSAs) and complete case analyses (CCAs) for all outcomes

Table S8a. *FSA: unadjusted and adjusted odds ratios (statin users vs statin non-users) for adverse events (any adverse event, any psychiatric symptom, anxiety, sleep disturbance, memory impairment, self-harm, suicidality, completed suicide, and all-cause mortality) at 2 months, 6 months, and 12 months*

		**FULL SET ANALYSIS** N 1,050,105					
		Unadjusted analysis			**Adjusted analysis*		
Outcome	*Time point*	*OR*	*99% CI*	*p*	*aOR*	*99% CI*	*p*
**All-cause mortality**	2mo	3.03	2.74, 3.35	<0.001	0.66	0.60, 0.72	<0.001
	6mo	3.43	3.14, 3.75	<0.001	0.66	0.61, 0.72	<0.001
	12mo	3.84	3.57, 4.13	<0.001	0.67	0.65, 0.73	<0.001
**Any adverse event**	2mo	1.11	1.05, 1.18	<0.001	0.93	0.91, 0.96	<0.001
	6mo	1.30	1.22, 1.38	<0.001	0.97	0.94, 0.99	0.008
	12mo	1.55	1.44, 1.66	<0.001	0.99	0.96, 1.03	0.637
**Any psychiatric symptom**	2mo	0.84	0.78, 0.89	<0.001	0.97	0.86, 1.09	0.494
	6mo	0.85	0.80, 0.90	<0.001	0.94	0.84, 1.04	0.117
	12mo	0.88	0.83, 0.93	<0.001	0.92	0.83, 1.02	0.045
**Anxiety**	2mo	0.57	0.53, 0.61	<0.001	1.07	0.92, 1.23	0.243
	6mo	0.55	0.52, 0.59	<0.001	1.07	0.93, 1.22	0.208
	12mo	0.54	0.51, 0.58	<0.001	1.05	0.92, 1.20	0.339
**Sleep disturbance**	2mo	1.12	0.98, 1.27	0.027	1.16	0.76, 1.77	0.361
	6mo	1.18	1.06, 1.32	<0.001	1.05	0.73, 1.50	0.753
	12mo	1.27	1.15, 1.40	<0.001	1.13	0.85, 1.50	0.254
**Memory impairment**	2mo	4.76	4.16, 5.45	<0.001	1.32	0.79, 2.20	0.159
	6mo	4.52	4.06, 5.03	<0.001	0.89	0.61, 1.31	0.448
	12mo	4.63	4.22, 5.09	<0.001	0.98	0.73, 1.31	0.841
**Self-harm**	2mo	0.29	0.21, 0.41	<0.001	0.76	0.50, 1.15	0.086
	6mo	0.27	0.21, 0.35	<0.001	0.78	0.56, 1.07	0.044
	12mo	0.27	0.22, 0.34	<0.001	0.82	0.62, 1.08	0.063
**Suicidality**	2mo	0.64	0.53, 0.76	<0.001	1.02	0.84, 1.23	0.827
	6mo	0.63	0.53, 0.74	<0.001	1.04	0.89, 1.21	0.546
	12mo	0.63	0.53, 0.76	<0.001	1.00	0.88, 1.14	0.940
**Completed suicide**	2mo	0.32	0.12, 0.87	0.003	0.72	0.18, 2.87	0.542
	6mo	0.27	0.12, 0.62	<0.001	0.54	0.18, 1.58	0.138
	12mo	0.33	0.18, 0.63	<0.001	0.82	0.35, 1.89	0.539
**Depressive episode remission**	2mo	1.09	0.86, 1.37	0.275	1.03	0.94, 1.13	0.335
	6mo	1.48	1.21, 1.81	<0.001	1.03	0.89, 1.20	0.521
	12mo	1.81	1.53, 2.15	<0.001	1.11	0.90, 1.38	0.139

Legend. For depressive episode remission an aOR>1 favours statin users, while for all outcomes an aOR<1 favours statin users. *Analyses adjusted for age; sex; BMI; type, severity, and year of diagnosis of depression; deprivation status; geographical area; smoking habits; alcohol use; ethnicity; comorbidities and concomitant medications use.

Table S8b. *CCA: unadjusted and adjusted odds ratios (statin users vs statin non-users) for adverse events (any adverse event, any psychiatric symptom, anxiety, sleep disturbance, memory impairment, self-harm, suicidality, completed suicide, and all-cause mortality) at 2 months, 6 months, and 12 months*

		**COMPLETE CASE ANALYSIS**					
		Unadjusted analysis *N 1,050,105*			**Adjusted analysis* *N 88,227*		
Outcome	*Time point*	*OR*	*99% CI*	*p*	*aOR*	*99% CI*	*p*
**All-cause mortality**	2mo	3.03	2.74, 3.35	<0.001	0.70	0.41, 1.19	0.083

	6mo	3.43	3.14, 3.75	<0.001	0.73	0.44, 1.23	0.083
	12mo	3.84	3.57, 4.13	<0.001	0.78	0.60, 1.02	0.016
**Any adverse event**	2mo	1.11	1.05, 1.18	<0.001	0.92	0.84, 1.02	0.032
	6mo	1.30	1.22, 1.38	<0.001	0.97	0.88, 1.06	0.333
	12mo	1.55	1.44, 1.66	<0.001	0.99	0.91, 1.09	0.873
**Any psychiatric symptom**	2mo	0.84	0.78, 0.89	<0.001	0.99	0.95, 1.02	0.172
	6mo	0.85	0.80, 0.90	<0.001	0.98	0.95, 1.01	0.100
	12mo	0.88	0.83, 0.93	<0.001	0.98	0.95, 1.01	0.124
**Anxiety**	2mo	0.57	0.53, 0.61	<0.001	1.04	0.99, 1.09	0.059
	6mo	0.55	0.52, 0.59	<0.001	1.03	0.98, 1.08	0.117
	12mo	0.54	0.51, 0.58	<0.001	1.02	0.98, 1.07	0.227
**Sleep disturbance**	2mo	1.12	0.98, 1.27	0.027	1.02	0.88, 1.18	0.761
	6mo	1.18	1.06, 1.32	<0.001	0.95	0.85, 1.06	0.192
	12mo	1.27	1.15, 1.40	<0.001	0.98	0.89, 1.07	0.496
**Memory impairment**	2mo	4.76	4.16, 5.45	<0.001	1.15	0.99, 1.34	0.013
	6mo	4.52	4.06, 5.03	<0.001	1.09	0.98, 1.21	0.037
	12mo	4.63	4.22, 5.09	<0.001	1.13	1.04, 1.23	<0.001
**Self-harm**	2mo	0.29	0.21, 0.41	<0.001	0.24	0.04, 1.24	0.025
	6mo	0.27	0.21, 0.35	<0.001	0.25	0.06, 1.01	0.011
	12mo	0.27	0.22, 0.34	<0.001	0.25	0.08, 0.73	0.001
**Suicidality**	2mo	0.64	0.53, 0.76	<0.001	1.10	0.75, 1.62	0.504
	6mo	0.63	0.53, 0.74	<0.001	0.98	0.68, 1.41	0.891
	12mo	0.64	0.53, 0.76	<0.001	0.95	0.69, 1.32	0.708
**Completed suicide**	2mo	0.32	0.12, 0.87	0.003	0.83	0.01, 68.13	0.911
	6mo	0.27	0.12, 0.62	<0.001	0.37	0.01, 13.43	0.479
	12mo	0.33	0.18, 0.63	<0.001	0.33	0.01, 9.89	0.399
**Depressive episode remission**	2mo	(N 87,996) 1.39	1.25, 1.53	<0.001	(N 26,245) 1.19	0.99, 1.43	0.013
	6mo	(N 25,695) 1.63	1.37, 1.94	<0.001	(N 6,318) 0.89	0.59, 1.36	0.505
	12mo	(N 16,880) 2.03	1.61, 2.56	<0.001	(N 3,743) 1.20	0.72, 2.01	0.352

Legend. For depressive episode remission an aOR>1 favours statin users, while for all outcomes an aOR<1 favours statin users.
*Analyses adjusted for age; sex; BMI; type, severity, and year of diagnosis of depression; deprivation status; geographical area; smoking habits; alcohol use; ethnicity; comorbidities and concomitant medications use.

S9. Comparison with aspirin (summary of regression analyses for aspirin), full set analysis (FSA)

All-cause mortality

2 months: no effect (p > 0.500)
6 months: no effect (p > 0.500)
12 months: negative effect (aOR 1.10, 99%CI 1.04 to 1.16, p < 0.001)

Any adverse event

2 months: negative effect (aOR 1.07, 99%CI 1.03 to 1.10, p <0.001)
6 months: negative effect (aOR 1.10, 99%CI 1.06 to 1.14, p <0.001)
12 months: negative effect (aOR 1.14, 99%CI 1.09 to 1.19, p <0.001)

Any psychiatric symptom

2 months: no effect (p > 0.003)
6 months: no effect (p > 0.009)
12 months: no effect (p > 0.069)

Anxiety

2 months: positive effect (aOR 0.90, 99%CI 0.84 to 0.96, p <0.001)
6 months: positive effect (aOR 0.91, 99%CI 0.86 to 0.97, p <0.001)
12 months: positive effect (aOR 0.91, 99%CI 0.85 to 0.96, p <0.001)

Sleep disturbance

2 months: no effect (p > 0.987)
6 months: no effect (p > 0.276)
12 months: no effect (p > 0.416)

Memory impairment

2 months: no effect (p > 0.883)
6 months: no effect (p > 0.780)
12 months: no effect (p > 0.563)

Self-harm

2 months: no effect (p > 0.270)
6 months: no effect (p > 0.671)
12 months: no effect (p > 0.421)

Suicidality

2 months: no effect (p > 0.917)
6 months: no effect (p > 0.611)
12 months: no effect (p > 0.399)

Completed suicide

2 months: no effect (p > 0.458)
6 months: no effect (p > 0.839)
12 months: no effect (p > 0.777)

Depressive episode remission

2 months: no effect (p > 0.907)

6 months: no effect (p > 0.683)

12 months: no effect (p > 0.406)

Legend. For depressive episode remission an aOR>1 favours aspirin users, while for all outcomes an aOR<1 favours aspirin users

S10. STROBE checklist

STROBE Statement—checklist of items that should be included in reports of observational studies

	Item No.	Recommendation	Page No.	Relevant text from manuscript
**Title and abstract**	1	(*a*) Indicate the study's design with a commonly used term in the title or the abstract	1	Mortality and adverse events associated with statin use in primary care patients with depression: a real-world, population-based cohort study
		(*b*) Provide in the abstract an informative and balanced summary of what was done and what was found	2-3	To assess the real-world safety of statins in depressive disorders. Statin use is associated with a lower all-cause mortality and adverse events in depression, which supports the safety of updated NICE guidelines for prescribing statins in this patients' group.
**Introduction**				
Background/rationale	2	Explain the scientific background and rationale for the investigation being reported	5	A clearer definition of the safety profile of statins is necessary to guide a truly informed prescription of these drugs (Cai et al. 2021), including in people with depression who are more at risk of premature CVD morbidity and all-cause mortality
Objectives	3	State specific objectives, including any prespecified hypotheses	5	Therefore, in this real-world, population-based cohort study, we investigate the mortality and adverse events associated with statin use vs non-use in a large,

				highly-characterised sample of primary care patients with depression followed up for 12 months.
**Methods**				
Study design	4	Present key elements of study design early in the paper	8	Outcomes of interest were explored with multivariable logistic regression models, clustered by general practices, to compute odds ratios (ORs) with 99% confidence intervals (99%CIs) for statin users vs statin non-users (i.e., between-subject design), adjusted (aOR) for the above potential confounders.
Setting	5	Describe the setting, locations, and relevant dates, including periods of recruitment, exposure, follow-up, and data collection	6-8	See headings: Database, Cohort construction, Exposure and comparison, Outcomes of interest
Participants	6	(*a*) *Cohort study*—Give the eligibility criteria, and the sources and methods of selection of participants. Describe methods of follow-up<br>*Case-control study*—Give the eligibility criteria, and the sources and methods of case ascertainment and control selection. Give the rationale for the choice of cases and controls<br>*Cross-sectional study*—Give the eligibility criteria, and the sources and methods of selection of participants	6	The study cohort was built according to the following inclusion/exclusion criteria to include a homogeneous population with first-episode depression (i.e., no treatment-resistant depression) followed up for 12 months.
		(*b*) *Cohort study*—For matched studies, give matching criteria and number of exposed and unexposed<br>*Case-control study*—For matched studies, give matching criteria and the number of controls per case	-	-
Variables	7	Clearly define all outcomes, exposures, predictors, potential confounders, and effect modifiers. Give diagnostic criteria, if applicable	6-8	See headings: Exposure and comparison, Outcomes of interest, Confounders
Data sources/ measurement	8*	For each variable of interest, give sources of data and details of methods of assessment (measurement). Describe comparability of assessment methods if there is more than one group	7	See heading: Outcomes of interest
Bias	9	Describe any efforts to address potential sources of bias	7	See heading: Confounders

Study size	10	Explain how the study size was arrived at	6	See headings: Database, Cohort construction

Continued on next page

Quantitative variables	11	Explain how quantitative variables were handled in the analyses. If applicable, describe which groupings were chosen and why	8	See heading: Analysis
Statistical methods	12	(*a*) Describe all statistical methods, including those used to control for confounding	7-8	See headings: Confounders, Analysis
		(*b*) Describe any methods used to examine subgroups and interactions	-	-
		(*c*) Explain how missing data were addressed	8	Multiple imputation by chained equations was employed for missing data: for each imputation, 10 imputed datasets were generated, and coefficient estimates across these were pooled using Rubin's rule (Rubin 1996) to calculate results for the primary full-set analysis (FSA, including imputed data).
		(*d*) *Cohort study*—If applicable, explain how loss to follow-up was addressed<br>*Case-control study*—If applicable, explain how matching of cases and controls was addressed<br>*Cross-sectional study*—If applicable, describe analytical methods taking account of sampling strategy	-	-
		(*e*) Describe any sensitivity analyses	8	As a sensitivity analysis, we reported results for the complete-case analysis (CCA), which does not include imputed data and only accounts for cases with no missing variables. Further, FSA data for statins were compared with those of the regression analyses for aspirin to probe whether results were non-specifically associated with another medication with comparable indication (i.e., prevention and treatment of CVD) in a similar population.
**Results**				
Participants	13*	(a) Report numbers of individuals at each stage of study—eg numbers potentially eligible, examined for eligibility, confirmed eligible, included in the study, completing follow-up, and analysed	9	The QResearch dataset included the electronic health records of 25,852,019 people registered with eligible general practices between

				1st of January 1998 and 15th of August 2020 for at least 12 months. After the application of inclusion/exclusion criteria, the study cohort comprised 1,050,105 patients with a diagnosis of first-episode depression: 90,094 statin users and 960,011 statin non-users.
		(b) Give reasons for non-participation at each stage	9	See Figure 1
		(c) Consider use of a flow diagram	9	See Figure 1
Descriptive data	14*	(a) Give characteristics of study participants (eg demographic, clinical, social) and information on exposures and potential confounders	10-12	See Table 1
		(b) Indicate number of participants with missing data for each variable of interest	10	Missing data, imputed for the FSA, are in Supplementary Material, S6. PHQ-9 values were the least available (81.26% missing), followed by alcohol consumption (48.68% missing), ethnic group (24.97% missing), and BMI (20.70% missing).
		(c) *Cohort study*—Summarise follow-up time (eg, average and total amount)	-	-
Outcome data	15*	*Cohort study*—Report numbers of outcome events or summary measures over time	13	Tables reporting the number of events for all outcomes per study group are in Supplementary Material, S7. A total of 21,384 (2.04%) people died, while most (67.34%, N 707,111) experienced at least one adverse event during the 12 months of study follow up. Respectively, we observed 262,110 (24.96%) records for any psychiatric symptom, 167,274 (15.93%) for anxiety, 19,291 (1.84%) for sleep disturbance, 8,524 (0.81%) for memory impairment, 6,707 (0.64%) for self-harm, 16,427 (1.56%) for suicidality, 561 (0.05%) for

				completed suicide, and 2,364 (0.23%) patients remitted from the depressive episode by study endpoint.<br>See Supplementary Material, S7
		Case-control study—Report numbers in each exposure category, or summary measures of exposure	-	-
		Cross-sectional study—Report numbers of outcome events or summary measures	-	-
Main results	16	(*a*) Give unadjusted estimates and, if applicable, confounder-adjusted estimates and their precision (eg, 95% confidence interval). Make clear which confounders were adjusted for and why they were included	13	On the FSA (N 1,050,105) adjusted model (Figure 2), statin use was associated with reduced all-cause mortality at all timepoints (aOR2mo 0.66, 99%CI 0.60 to 0.72; aOR6mo 0.66, 99%CI 0.61 to 0.72; aOR12mo 0.67, 99%CI 0.65 to 0.73). A lower number of people experienced any adverse events at two and six months (respectively aOR2mo 0.93, 99%CI 0.91 to 0.96; aOR6mo 0.97, 99%CI 0.94 to 0.99), but not at 12 months (aOR12mo 0.99, 99%CI 0.96 to 1.03) was noted in statin users. No differences were identified at any timepoint for any psychiatric symptom (range aOR2-12mo 0.92-0.97), anxiety (range aOR2-12mo 1.05-1.07), sleep disturbance (range aOR2-12mo 1.05-1.16), memory impairment (range aOR2-12mo 0.89-1.32), self-harm (range aOR2-12mo 0.76-0.82), suicidality (range aOR2-12mo 1.00-1.02), completed suicide (range aOR2-12mo 0.54-0.82), or depression remission (range aOR2-12mo 1.03-1.11).<br>See Figure 2, Supplementary Material, S8
		(*b*) Report category boundaries when continuous variables were categorized	-	-

	(*c*) If relevant, consider translating estimates of relative risk into absolute risk for a meaningful time period	-	-

Continued on next page

Other analyses	17	Report other analyses done—eg analyses of subgroups and interactions, and sensitivity analyses	14-15	In statin users, results from the sensitivity analysis on the CCA (N 88,227) adjusted model, also showed lower all-cause mortality (range aOR2-12mo 0.70-0.78) and any adverse events (range aOR2-6mo 0.92-0.97), but with wide confidence intervals that crossed the line of no effect. Consistently with the FSA, any psychiatric symptom (range aOR2-12mo 0.98-0.99), anxiety (range aOR2-12mo 1.02-1.04), sleep disturbance (range aOR2-12mo 0.95-1.02), suicidality (range aOR2-12mo 0.95-1.10), completed suicide (range aOR2-12mo 0.33-0.83), and depression remission (range aOR2-12mo 0.89-1.19) were not different. However, a decrease in self-harm episodes (aOR12mo 0.25, 99%CI 0.08 to 0.73) and higher memory impairment (aOR12mo 1.13, 99%CI 1.04 to 1.23) in statin users at 12 months only was observed. Tables including all analyses (FSAs and CCAs, both adjusted and unadjusted)

				are in Supplementary Material, S8. Outcomes for the sensitivity analysis on aspirin (Supplementary Material, S9) only partially overlapped with those of statins: aspirin use was associated with worse all-cause mortality at 12 months (aOR12mo 1.15, 99%CI 1.07 to 1.24) and adverse events at all timepoints (aOR2mo 1.07, 99%CI 1.07 to 1.11; aOR6mo 1.12, 99%CI 1.07 to 1.17; aOR12mo 1.18, 99%CI 1.12 to 1.24), though also less anxiety over follow up (aOR2mo 0.90, 99%CI 0.84 to 0.96; aOR6mo 0.91, 99%CI 0.86 to 0.97; aOR12mo 0.91, 99%CI 0.85 to 0.96). See Supplementary Material S8, S9
**Discussion**				
Key results	18	Summarise key results with reference to study objectives	16	This study examined an array of safety outcomes associated with statins in a large cohort of 1,050,105 patients with a new diagnosis of depressive disorder followed up for 12 months in real-world conditions. The FSA

adjusted model showed that statin use, compared to non-use, was associated with lower all-cause mortality, and lower or at worst comparable adverse events over the follow-up period. Results differed from those of the sensitivity analysis on aspirin use, which instead displayed higher mortality rates and adverse events compared to aspirin non-use. No differences for total psychiatric symptoms, anxiety, sleep disturbance, memory impairment, self-harm, suicidality, completed suicide, or depressive episode remissions were observed between statin users and non-users. A sensitivity analysis of the 88,227 cases with no missing data (CCA) supported the latter lack of associations but for lower self-harm and higher memory impairment at 12 months, while it did not identify any difference for mortality and total adverse events.

Limitations	19	Discuss limitations of the study, taking into account sources of potential bias or imprecision. Discuss both direction and magnitude of any potential bias	18-19	See heading: Limitations
Interpretation	20	Give a cautious overall interpretation of results considering objectives, limitations, multiplicity of analyses, results from similar studies, and other relevant evidence	18-19	See heading: Limitations
Generalisability	21	Discuss the generalisability (external validity) of the study results	19	On the other hand, we could conduct a real-world assessment of the adverse outcomes associated with statin use in a large population of people with depression – including those with multiple comorbidities and co-occurring treatments oftentimes excluded from clinical trials, thus increasing the external validity of our results
**Other information**				
Funding	22	Give the source of funding and the role of the funders for the present study and, if applicable, for the original study on which the present article is based	2	See heading: Funding

*Give information separately for cases and controls in case-control studies and, if applicable, for exposed and unexposed groups in cohort and cross-sectional studies.

Note: An Explanation and Elaboration article discusses each checklist item and gives methodological background and published examples of transparent reporting. The STROBE checklist is best used in conjunction with this article (freely available on the Web sites of PLoS Medicine at http://www.plosmedicine.org/, Annals of Internal Medicine at http://www.annals.org/, and Epidemiology at http://www.epidem.com/). Information on the STROBE Initiative is available at www.strobe-statement.org.

Supplementary references

1. De Crescenzo F, Garriga C, Tomlinson A, Coupland C, Efthimiou O, Fazel S, et al. Real-world effect of antidepressants for depressive disorder in primary care: protocol of a population-based cohort study. Evid Based Ment Health. 2020;23(3):122-6.
2. Hippisley-Cox J, Vingradova J, Coupland C, Pringle M. Comparison of key practice characteristics between general practices in England and Wales and general practices in the QRESEARCH database NHS information centre2005 [Available from: https://www.qresearch.org/Public_Documents/Characteristics%20of%20QRESEARCH%20practices%20_database%20version%208_%20v1.0.pdf.
3. Coupland C, Dhiman P, Morriss R, Arthur A, Barton G, Hippisley-Cox J. Antidepressant use and risk of adverse outcomes in older people: population based cohort study. Bmj. 2011;343:d4551.
4. Coupland C, Hill T, Morriss R, Moore M, Arthur A, Hippisley-Cox J. Antidepressant use and risk of adverse outcomes in people aged 20-64 years: cohort study using a primary care database. BMC Med. 2018;16(1):36.
5. De Giorgi R, De Crescenzo F, Cowen PJ, Harmer CJ, Cipriani A. Real-world outcomes of concomitant antidepressant and statin use in primary care patients with depression: a population-based cohort study. BMC Med. 2023;21(1):424.
6. Chevance A, Ravaud P, Tomlinson A, Le Berre C, Teufer B, Touboul S, et al. Identifying outcomes for depression that matter to patients, informal caregivers, and health-care professionals: qualitative content analysis of a large international online survey. Lancet Psychiatry. 2020;7(8):692-702.
7. Law M. Having too much evidence (depression, suicide, and low serum cholesterol). Bmj. 1996;313(7058):651-2.
8. You H, Lu W, Zhao S, Hu Z, Zhang J. The relationship between statins and depression: a review of the literature. Expert Opin Pharmacother. 2013;14(11):1467-76.
9. Tuccori M, Montagnani S, Mantarro S, Capogrosso-Sansone A, Ruggiero E, Saporiti A, et al. Neuropsychiatric adverse events associated with statins: epidemiology, pathophysiology, prevention and management. CNS Drugs. 2014;28(3):249-72.
10. Alghamdi J, Matou-Nasri S, Alghamdi F, Alghamdi S, Alfadhel M, Padmanabhan S. Risk of Neuropsychiatric Adverse Effects of Lipid-Lowering Drugs: A Mendelian Randomization Study. Int J Neuropsychopharmacol. 2018;21(12):1067-75.
11. De Giorgi R, Rizzo Pesci N, Quinton A, De Crescenzo F, Cowen PJ, Harmer CJ. Statins in Depression: An Evidence-Based Overview of Mechanisms and Clinical Studies. Front Psychiatry. 2021;12:702617.
12. Samaras K, Makkar SR, Crawford JD, Kochan NA, Slavin MJ, Wen W, et al. Effects of Statins on Memory, Cognition, and Brain Volume in the Elderly. J Am Coll Cardiol. 2019;74(21):2554-68.
13. Kroenke K, Spitzer RL, Williams JB. The PHQ-9: validity of a brief depression severity measure. J Gen Intern Med. 2001;16(9):606-13.
14. Vinogradova Y, Coupland C, Brindle P, Hippisley-Cox J. Discontinuation and restarting in patients on statin treatment: prospective open cohort study using a primary care database. Bmj. 2016;353:i3305.
15. Townsend P, Phillimore P, Beattle A. Health and deprivation: inequality and the North. London: Croom Helm; 1988. 236 p.
16. Rubin DB. Multiple Imputation after 18+ Years. Journal of the American Statistical Association. 1996;91(434):473-89.
17. StataCorp. Stata Statistical Software: Release 17. College Station, TX: StataCorp LLC.; 2021.
